# Editorial Notes

**Published:** 1904-09

**Authors:** 


### Editorial Notes.

**British Medical Association.**

THE seventy-second annual meeting of the British Medical Association, which was held in Oxford, was beyond question one of the most successful meetings in the memory of members of the Association, as it was in point of numbers much the largest yet recorded. It was remarkable also for the persistence and enthusiasm with which, in spite of all the counter-attractions of that ancient and glorious seat of learning, and of the diversions, entertainments and receptions arranged by the members of the University and by the citizens, a quite unusually large proportion of the members who were visiting Oxford steadily pursued the actual business of the meeting in the various sections. Not only was the occasion distinguished by the presence and participation in the sectional meetings of a considerable number of eminent foreign visitors, and of an exceptionally numerous gathering of the recognised leaders of thought and investigation in medical science in our own country and the colonies, but it was also rendered memorable by the great importance and originality of the new work brought forward in many of the sections. The above views are those of a writer in *Nature* (August 4th, 1904, p. 332), and we quote them, as coming from a scientific rather than a professional standpoint, to show that the annual meeting of the Association has not yet degenerated into an annual picnic.

The fair city of Oxford presents so many attractions that the meetings might well have taken second place in the minds of the visitors, for whom the civic and the University authorities did everything possible to further the credit of the city and the enjoyment of its guests. The medical members were not allowed to linger too long on the

> "Strange enchantments of the past
> And memories of the days of old,"

but the president's address breathed the spirit of the Oxford of the present day, for so many and such rapid changes have occurred that the revival of the medical school is only on a line with the complete change which has come over the city during the last few decades. The president's reference to the earlier benefactors of the University recalled to his hearers that ancient past, to which so many speakers subsequently alluded, more particularly Professor Osler, who, speaking in the hall of Christ Church, made especial allusion to John Locke, whose portrait is hanging on the walls, and who was distinguished not more by his intellectual insight than by his rational piety, love of truth for its own sake, and manly sympathy with civil and religious liberty. We, in the West of England, may well recall the fact that Locke was born at Wrington, and that he has been called the father of modern inductive psychology and the Socrates of England. The addresses in medicine and surgery have been fully reported. The former is likely to be of great service in the work of sanitary organisation which the Association is endeavouring to perform.

As regards the work of the sections, it is difficult to select any points for comment in a brief notice such as this must necessarily be. It was anticipated that a discussion on chloroform anæsthesia would attract a large attendance; accordingly the meeting was held in the hall of Balliol College, and was presided over by Professor Gotch, F.R.S.

The debate opened by Professor Osler in the medical section was rendered memorable by the following observation, which is in advance of anything hitherto believed, when he stated that "in the absence of evidence in any case of pleurisy of a pneumonic or pyogenic origin it should be assumed, for the purposes of treatment, that it was tuberculous."

Papers on Giants and Dwarfs and Ateleiosis were read, and the presence of a remarkable group of dwarfs excited much interest. Recent papers on this subject are referred to in our *Progress* (page 249), and a paper on Infantilism by Dr. Cecil Williams is found on another page (*vide* page 247).

A discussion on the control of the milk supply was opened by Dr. Newman, whose conclusion was that the demand for reform must come from the consumer, who appeared to wish to have good milk at a price for which only bad milk could be procured. A paper on this subject by Dr. Brickdale (page 196) is of much interest in this connection.

The Pathological Museum was one of which the local committee might well be proud. Photographic, stereoscopic, radiographic, and microscopic preparations were very numerous, and many of them most instructive, the demonstration of the cellular changes occurring in malignant growths being of great interest at the present time. The *British Medical Journal* reminds us that Oxford saw the institution of an annual museum, and Oxford has now set a standard of excellence in illustration of the more recent developments of medical science which will be exceedingly difficult to maintain.

The annual exhibition of foods, drugs, instruments, and sanitary appliances, held in the Examination Schools, was very conveniently placed, and the exhibits were of the usual varied character. They are for the most part familiar, but some of them, which have not previously received comment in our pages, will duly receive attention in our Notes on Preparations for the Sick.

The following notes, from one of the representatives of the Bath and Bristol Branch, show that the new constitution of the Association is working out satisfactorily:—

The annual meeting of the representatives, as in the previous year, showed considerable business ability on the part of its members, and a refreshing, and perhaps not altogether expected, absence of irrelevant verbosity. The establishment of these meetings of representatives must be regarded as a decided success in combining the orderly conduct of the business of the Association with representation of the members on a democratic basis, and in providing a suitable opportunity for the discussion of matters of general medical importance. No small share in this success is to be attributed to the tact and capacity of the chairman, Sir Victor Horsley, who deserves great praise for his conduct of the meetings, and who, apart from the actual duties of chairmanship, brought to it a wide knowledge of medico-political matters which was of great service to the meeting.

That the chairman is a member of the General Medical Council is of especial advantage in this respect, and it is much to be hoped that Sir Victor will retain the chairmanship for another year, if not longer. The chief subjects dealt with by the meeting have already been published in the *British Medical Journal*. Brief mention may be made of the discussion of Medical Defence, on which no direct vote was taken, as the returns sent in from the divisions were not complete. The general impression left by the discussion is that given in the Editorial Notice (August 6th) in the *British Medical Journal*, that the undertaking of Medical Defence by the Association should be combined with some form of amalgamation with the societies which already exist for that purpose. Some general principles of Hospital Reform, formulated by the Hospitals' Committee, were adopted by the meeting as a basis for further action in which the co-operation of laymen interested in hospital work should be obtained, and the attainment of a moderate measure of reform, on all hands acknowledged to be greatly needed, thereby rendered more probable. Amongst other matters the reader will find some useful facts with regard to the furnishing of information to coroners by medical men in the Supplement to the *Journal* of August 6th.

It was resolved to take steps to correct the omission of provision in the Midwives Act for the payment of a fee to a medical man called to render assistance; and the majority of the meeting was in favour, quite rightly as we think, of the registration of nurses. It seems to us that the objection often made to registration—namely, that it will confer a recognised status on incompetent nurses and so enable them to compete on equal terms with properly trained and efficient ones—is quite unfounded. No nurse, any more than a doctor, will get patients by being on a register; success in her avocation will depend, as before, on her ability as a nurse, but the Act will ensure a certain fixed minimum of training, and exercise a restraint on the vicious and unscrupulous, who are to be found in all professions, and in these two ways protect the public. Among minor matters a resolution was moved which will enable, when carried into effect, branches or divisions to help out of their surplus funds medical benevolent societies, or the widow or family of a brother practitioner. This was the custom of many branches under the old constitution of the Association, and in our opinion is a highly desirable one, for care of its less fortunate members is a source of strength to an Association such as ours.

**Hospital Administration.**

THE question of the form and amount of hospital abuse is a vexed one even amongst members of the medical profession, and it is not therefore a matter of surprise that the general public has shown a scant and tardy interest in the subject.

The difficulty of dealing with so complex a thing as the distribution of medical charity, and the desire, both of the profession and the public, not to treat the deserving poor with harshness, are largely responsible for the gradual growth of an abuse which has now reached considerable proportions.

A sub-committee of the Bath and Bristol Branch of the British Medical Association, appointed in 1895 to inquire into this subject, found that no less than 356 per 1,000 of inhabitants in Bristol were in receipt of gratuitous medical relief. These figures are but a type of what is happening all over the country, and it is obvious that the funds of the medical charities are thereby unnecessarily depleted. A further result is that the overcrowding of the various departments engages much of the time and attention of the doctors which should be given to the really deserving cases that are apt to be crowded out; and the pauperisation of a large part of the community is slowly but surely being accomplished by the very means which were primarily intended for their help in time of stress.

One of the newer and subtler forms of abuse, which has not attracted much attention, because it appears superficially to be free from objection, is brought about by workmen's subscriptions to the medical charities. Such moneys are usually obtained by the formation of a sort of club amongst the workmen of certain firms—each employé putting aside a penny per week of his wages—and with the sum annually collected notes are "purchased" at the various medical charities. On comparing the number of notes so issued with the cost of the attendance of the patients if all the notes were used, we find that in one institution in Bristol there would be a loss of about £5,000 per annum.

In so far as the notes issued are devoted to the help of those who could not afford to pay for proper medical attendance this loss need not be objected to; but it seems probable that many of the employés who use these notes are in receipt of sufficient wages to allow them to pay for medical advice.

The existence of the note system—which we think might be abolished if some means of checking abuse existed—is largely answerable for this increasing and unwarrantable form of hospital expenditure.

We refrain from further comment on many other aspects of this question, as we print below a paper from Miss Sturge which covers the chief points so admirably, and has perhaps greater weight because the writer approaches the subject from the lay point of view; but it should be remembered that what is true of Bristol is true of other parts of the country, so that the needless expenditure of time and money and the pauperisation of the lower classes is general.

We are glad to publish a paper written by a well-known Bristol philanthropist, who is interested not only in our poor and the hospitals, but is also one of the leaders in the work of charity organisation. Miss Elizabeth Sturge writes on "The Misuse of Medical Charities," and her views are such that we may almost adopt them as those current in the medical profession at the present time.

**The Misuse of Medical Charities.** By Miss Elizabeth Sturge.—No one can question the beneficence of the work carried on by our great medical institutions, a work which becomes every year more effective as science advances, and places at the disposal of doctors means but yesterday unthought of for the cure of disease and the alleviation of suffering. It is in hospitals that new discoveries are first applied, and the whole population benefits by their growth in completeness and efficiency.

But here, as elsewhere, development brings with it the need of organisation. There is always a danger, if one aspect only of a subject is considered, that the good done in one direction may be partially neutralised by evil unwittingly done in another; and this is the case if we consider the hospital question from the standpoint of disease alone, and not also as part of a great social problem—the problem of helping those in need without robbing them of their most valuable heritage, the power and inclination to help themselves. Inevitably our lives and habits are influenced by the institutions under which we live. This is a law from which there is no escape, and applies, as the history of the poor law tells us, with fatal force to all schemes for providing the poor, without certain safeguards, with the necessaries of daily life at the public expense. That this law applies to the inevitable contingency of sickness is shown by the fact that provident dispensaries cannot be carried on in the neighbourhood of large hospitals, though they succeed in localities sufficiently distant, or in towns where, as at Manchester, a considerable number of the hospitals have a system of inquiry which restricts the out-patients to persons whose means fall below a certain standard. Such a rule is not found so necessary in the case of in-patients, reluctance to enter an institution being usually a sufficient guarantee against abuse.

All doctors who have had experience in an out-patient department know that many persons appear there who could well afford to pay a moderate fee. In 1895 the Council of the Bath and Bristol Branch of the British Medical Association appointed a sub-committee to inquire into the question of hospital abuse in Bristol. The report of that committee showed that at the Royal Infirmary out of 1,000 cases taken at random 235 might be so described. It is a serious thing that such a misapplication of charity should go on—there is no reason to believe it worse here than elsewhere—not only because of the demoralising effect on these well-to-do people and their friends of receiving gratis what they can afford to pay for, but also because of the unfairness of such abuse to the public and to the hospital managers, who struggle incessantly against an ever-accumulating load of debt. There are signs that the "intolerable strain" may lead to a demand for the municipalisation of hospitals, and should this be the case, it is evident some means would have to be adopted for deciding who should be permitted to benefit by what would be in effect a large extension of the poor law.

This is not, however, the only point of view from which we must consider this subject. There is the question of justice to the medical profession. Doctors are some of the most generous of men, but are not as a class among the richest. They willingly give their services to help the really poor, and in private show much kindness, which the world never hears of, to patients whose circumstances preclude any return; but it is not fair, and the public has no right to expect, that they should have to attend people gratuitously who can afford to pay a fee.

The problem is no doubt a difficult one to solve. The demands of illness are imperious, and surgery requires such skill as can only be gained in hospitals; but the experience of Manchester goes far to show that the difficulty can be overcome. If it is once understood that a wage standard is in force, and that the circumstances of out-patients will be inquired into, the difficulty tends to disappear of itself. A great impetus is at once given to provident dispensaries and sick clubs, and a large number of the cases cease to attend. In Manchester the number of unsuitable applicants fell from 42.32 in 1875, the year in which the system of inquiry was begun, to 5.05 in 1903. Meantime, a Provident Dispensary Association established branches in ten districts, with an aggregate membership of 18,620, and this number would no doubt be largely increased were all the hospitals to adopt the same system of inquiry.

One of the chief obstacles in the way of systematic action on these lines is the fact that most hospital authorities use the out-patient figures as a lever for begging appeals, and would probably view with disfavour any diminution in numbers. In the present state of public opinion there is some force in this difficulty. The public needs to be taught that large numbers do not imply the best work. In an out-patient department they certainly show that the doctors are overworked, the ablest men in the profession wasting time in seeing cases of no medical or surgical importance; and, from the patients' point of view, that persons seriously ill are worn-out with hours of waiting while the trivial cases are taking their turn. It would be an immense advantage if the benefits of the hospital could be reserved for serious cases, the provident dispensary and club doctors sending any for whom another opinion was desirable to the hospital specialist.

It may be objected that to restrict the attendance of out-patients would be to inflict hardship on the poor, because the unskilled working man cannot afford even low fees. To this it may be replied that in localities at a distance from hospitals, such as the newer districts on the north and east of Bristol, it is found that far more is spent by poor people in fees than *à priori* would appear to be likely; but admitting the difficulty of single fees, the system of insurance can be applied to this as to many of the other contingencies of life. Medical attendance forms part of the benefits conferred by all the best clubs, and for a weekly payment of a few pence a whole family can secure it in a provident dispensary.

In London the subject has long been under consideration, but the hospitals have been very slow to adopt any system for restricting the enormous number of their out-patients—for advertising purposes a valuable asset. At several of them, however, the need for doing so is recognised, and an official (usually a lady) has been appointed under the title of

"Almoner," whose duty it is to see the out-patients, find out who are ineligible for gratuitous treatment, and see whether the necessities of the others are met by attendance at the hospital, or whether they have other pressing needs such as that for food or convalescent aid. To secure such help the work is carried on in co-operation with the Charity Organisation Society, to which these cases are referred. The presence of such an official not only tends to keep away unsuitable applicants but aids the humane work of the hospital by securing where possible the good home conditions without which medical treatment is of little avail.

The committee which carried out the inquiry referred to at the Bristol Royal Infirmary found that about £700 per annum could be saved there if unsuitable cases were refused. Surely as public opinion grows more enlightened, and the difficulty of maintaining hospitals becomes more acute, an improvement like this will be called for, for such a change would be at once a measure of economy for the managers, of humanity to the poorer patients, and of justice to the doctors and general public.

